# Participation in formal learning activities of older Europeans in poor and good health

**DOI:** 10.1007/s10433-016-0371-6

**Published:** 2016-04-18

**Authors:** Stanisława Golinowska, Agnieszka Sowa, Dorly Deeg, Marco Socci, Andrea Principi, Ricardo Rodrigues, Stefania Ilinca, Henrike Galenkamp

**Affiliations:** 1 Collegium Medicum Jagiellonian University, Kraków, Poland; 2 Institute of Labour and Social Studies (IPiSS), Warsaw, Poland; 3 Center for Social and Economic Research (CASE), Warsaw, Poland; 4 The EMGO Institute for Health and Care Research, VU University Medical Center, Amsterdam, The Netherlands; 5 National Institute of Health and Science on Ageing (INRCA), Ancona, Italy; 6 European Centre for Social Welfare Policy and Research, Vienna, Austria

**Keywords:** Older people, Human capital, Learning activities, Labour market activity, Morbidity, Active ageing, Successful ageing

## Abstract

Little attention has been given to the involvement in formal learning activities (FLA) in the older population when considering different health statuses. The aim of this study is to explore the extent to which possible predictors (derived from previous research as well as a conceptual model) of FLA differ for older people in poor and good health. Data are used from SHARE 2010/2011 for the 50+ populations in 16 European countries. Poor health is defined as self-report of having two or more chronic diseases assessed by a medical doctor, i.e. multimorbidity. Possible predictors of learning activities represent individual characteristics: functional limitations, demography (age, gender, marital status and household size), human capital (achieved level of education), employment, income and participation in other social activities. To assess the predictors of FLA, logistic regression models are used and average marginal estimates are compared across groups. In addition to multimorbidity, labour market activity is used as a grouping variable. The average participation of individuals in the group with multimorbidity was nearly 50 % lower than that in the group in good health (6.5 vs. 13.3 %). Regardless of multimorbidity, human capital proved to be significant predictors of FLA, especially in those active on the labour market. However, the associations were weaker in the multimorbidity group. Also, significant associations were observed of other types of social activities, in particular cultural and leisure activity and volunteering, with FLA. This study suggests that similar factors are predictors of FLA in older people with and without multimorbidity.

## Introduction

Education is the basic domain of human capital. The OECD has proposed a broad definition of human capital as the knowledge, skills, competencies and attributes acquired by an individual’s learning activities that facilitate the creation of personal, social and economic well-being (OECD 2001). It can be measured by years in school or by level of education (Hanson 2008; Liu and Fraumeni 2014). Human capital brings a lot of benefits; intrinsic and external (for society). It has a positive impact on economic status through increasing job opportunities and chances for higher earnings (Becker 2009). It improves social status and social well-being (Marmot 2005; Lochner 2011; OECD 2011), changes preferences and behaviours (Arrow 1997), and focuses the individual on future over current consumption (Becker and Mulligan 1997). In this article adult educational activity known as life-long learning is considered with a focus on formal (organised—Heisel et al. 1981) learning activity of older people. These are carried out in various organisational settings and do not include self-education. It is assumed that formal learning activity (FLA) of older people is a factor increasing social contact, social integration and the willingness to cooperate—important elements of active and successful ageing.

Learning activities of older people play a role in the framework of three mostly elaborated and affiliated concepts: active ageing, healthy ageing and successful ageing. The concept of active ageing (WHO 2002, p. 12) stresses ‘opportunities for physical, social and mental well-being throughout the life course in order to extend healthy life expectancy, productivity and quality of life in older age’. The concept of successful ageing considers the phenomenon of the ‘low probability of diseases and disease related disabilities, high cognitive and physical functioning and active engagement with life’ (Rowe and Kahn 1998; Minkler and Fadem 2002, p. 229). The concept of healthy ageing is interpreted as the ‘ability to continue to function mentally, physically, socially and economically as the body slows down its processes’ (Hansen-Kyle 2005, p. 46). Learning activities at an older age are embedded in the concept of successful ageing, being one of the types of social activities and social connections that are added to the broader meaning of health status and health risks in older age.

The health status of older people is generally characterised by the presence of one or more diseases. Mostly, these diseases are chronic and do not exist as a single health problem, but rather as multimorbidity, i.e. the co-existence of two or more chronic diseases. Epidemiological studies indicate that the prevalence of multimorbidity at older ages is assessed at more than 50 %; for example, it has been reported to range from 55 to 98 % (Marengoni et al. 2011) or 60–80 % (Salive 2013). This article proposes the hypothesis that the formal learning activity of older adults differs depending on the presence of multimorbidity.

Research points to human capital factor that might increase the probability of participation in learning courses in older age and to mental or cognitive health problems as factors potentially decreasing participation (Baltes 1993; Aartsen et al. 2002). In this study, we examine whether these predictors show differences between multimorbidity groups. Given that formal learning activity in adulthood is often stimulated by labour market activity (Holford et al. 2008; Mitchell 2007; Martin et al. 2009), and thus may be of a different nature than learning activity of older people who are no longer active on the labour market (Withnall 2000), this study addresses predictors of formal learning activity by making a distinction between being active on the labour market or not.

## Predictors of formal learning activities generally and specifically in older age from previous research

Research on the dependencies between the level of education and engagement in adults’ formal learning activity indicates a strong positive relation (Findsen and Formosa 2011). Generally, people with higher level of education display a greater propensity for further learning (Graney 1980; Tuckett and Sargant 1999; Mitchell 2007; Tuckett and Aldrige 2009). This relation is strengthened by employers’ preferences towards employees’ formal learning activity. Employers are more likely to invest in the formal learning activity of individuals with a higher education (OECD 2013). However, the formal learning activities (FLA) of workers are not necessarily conducive to social engagement. Borgonovi and Miyamoto (2010) interpret formal learning activity more as an expression of competitive labour market behaviour and individualism than as a factor contributing to social capital development. In older age work-related courses are still present, however in a lower scale than earlier. American statistical data from Council of Education (2007) indicates that only 5 % of people 65+ participate in work-related courses, while in personal interest courses—19 %. Authors of the British study on learning activity of older people (Shiela and Soulsby 1998) underline that many individuals do not feel the need to participate in FLA (Withnall 2002), preferring informal activities and self-education, which might include watching TV, reading newspapers, going to the library and acquiring knowledge via computer (Holford et al. 2008). However informal learning is not always perceived as learning, rather as typical activities associated with higher well-being (Jenkins and Mostafa 2012).

A qualitative study on the determinants of undertaking learning activities among older people in Great Britain suggests that past participation in the labour market and employment history positively impact the possibility of formal life-long learning past retirement age. The study found that the majority of those participating in learning courses later in life have been actively engaged in acquiring new competences and learning in the past (Withnall 2002). Occupation is another important factor, correlated with education and past history of learning. Individuals working in the past as semi-professionals or professionals are more likely to engage in formal learning post-retirement age. The amount of time since retirement is another important factor for formal learning activity (i.e. not more than 10 years was an optimal period for engagement in FLA).

An extended above-retirement-age working life is a significant predictor of formal learning in older age. However, labour market activity in older age depends on institutional rules concerning retirement age, incomes in older families, skills of the older generation and employers’ needs. Studies (e.g. from Finland—von Werder and Thum 2013) show that if older people are still professionally active, their interest in learning activities is high. Better-educated workers stay longer in employment and continue to participate in learning courses.

Low income and poor living conditions might be among the factors that restrict the formal learning activity of older people. The OECD (2013) finds financial or organisational barriers to engagement in life-long learning, especially for the older population.

Additionally, other activities, such as family obligations might be an obstacle to formal learning activity of older people. The chances for individuals involved in the provision of care to initiate learning courses are lower due to the opportunity costs and limited time left for such activities. However, Alex Withnall found that the impact of caring as a barrier to learning might be overcome by the assurance of adequate means of transportation: either by car or easily available public transportation. Similar to informal care provision, volunteering was seen as an obstacle to formal learning, simply because of the lack of time or need to engage in both types of activities (Withnall 2002).

## Formal learning activities later in life and health

Research on the FLA of older people indicates that seniors are active under two main conditions: when they are healthy or when they are well educated. In economic literature, good health is considered as a growth factor, similar to the level of education. Michel Grossman treats health as a complementary factor to human capital and indicates the mutual relationship of education and health (Grossman 1972, 2000, 2006; Leigh 1983; Grossman and Kaestner 1997; Brunello et al. 2011). Indeed, several studies indicate (e.g. Sloane-Seale and Kops 2010) that physically and mentally healthy people are socially active in a variety of activities, whereas in particular long-term chronic disease and multimorbidity are the main reasons for an inactive life at advanced age.

The FLA of older people with poor health have not yet been studied even though SOC theory (Selection, Optimization, and Compensation) creates framework for it (Baltes et al. 1999). It suggests that people have a certain age-related plasticity, and all the time make selection of life goals, adjust and optimise methods to achieve these goals and find compensation if their achievement is too difficult. Because the health conditions of older people are generally worse and multimorbidity is not an exception but rather a frequent condition at later stages of life (Marengoni et al. 2011; Sinnige et al. 2013), it is important to investigate what kind of formal learning activity predictors can be found in older people in poor health.

Since formal learning activity of older people often takes place in the context of labour market activity, it has been additionally examined if the predictors of formal learning activity in those who are active on the labour market differ from those who are not.

## Methods

This study concentrates on identifying the predictors of formal learning activity at older age among people with and without multimorbidity and with active and inactive labour market status. Potential predictors include demographic factors, health conditions and socio-economic status as well as other social participation activities.

### Sample description

The data used are from the Survey of Health, Ageing and Retirement in Europe (SHARE) conducted during 2010–2011 (Wave 4, Release 1.1.1). SHARE is a cross-national survey study covering individuals aged 50+ in 16 European countries. For FLA data were available from 57,391 individuals what constitutes a research sample (out of the total SHARE sample of 58,498 individuals).

Almost one in four respondents suffers from multimorbidity (see Table 1). Over half of the sample is female. A large fraction of the sample is under 65; however, every fifth person is aged 75+. In the 75+ age group, multimorbidity is two times higher than no multimorbidity. In the 50–64 age group, every third person suffers from multimorbidity. More than half live in households consisting of two members, while approximately one-fifth live in single households and almost one-fourth in households with three or more members. Approximately 21 % of respondents in the sample have obtained a higher education, while about 40 % have either a primary or secondary education level. More than half of the sample are retirees and 27 % are employed or self-employed. Unemployment or medical leave occur infrequently. When social activities are in question, every fourth person is involved in sports or club activities or is providing some type of care. Volunteering, religious activities and organised learning activities are less common.

**Table 1:** Sample characteristics: total sample and by morbidity status

Variables	Categories	Sample *N* = 57391		Less than 2 morbidities *N* = 44122		2 morbidities or more *N* = 13269	
		*N*	%	*N*	%	*N*	%
1 ADL+		6663	*11.62*	3021	*6.85*	3642	*27.46*
Poor mental health		23,933	*42.64*	15,820	*36.57*	8113	*63.06*
Dementia		700	*1.22*	367	*0.83*	333	*2.51*
Demographics							
Gender	Male	24,904	*43.39*	19,442	*44.06*	5462	*41.16*
	Female	32,487	*56.61*	24,680	*55.94*	7807	*58.84*
Age	50–64	27,241	*48.48*	23,308	*54.21*	3933	*29.81*
	65–74	16,631	*29.60*	12,346	*28.71*	4285	*32.47*
	75+	12,318	*21.92*	7341	*17.07*	4977	*37.72*
Marital status	Single	3164	*5.59*	2517	*5.79*	647	*4.92*
	Married	40,390	*71.38*	32,020	*73.70*	8370	*63.70*
	Divorced	4948	*8.74*	3776	*8.69*	1172	*8.92*
	Widowed	8082	*14.28*	5132	*11.81*	2950	*22.45*
Household size	Single	11,839	*20.63*	8164	*18.50*	3675	*27.70*
	2 persons	31,895	*55.57*	24,601	*55.76*	7294	*54.97*
	3 persons+	13,657	*23.80*	11,357	*25.74*	2300	*17.33*
Human capital							
Education	Primary	21,314	*39.04*	15,233	*36.17*	6081	*48.75*
	Secondary	21,794	*39.92*	17,291	*41.05*	4503	*36.10*
	Higher	11,486	*21.04*	9596	*22.78*	1890	*15.15*
Socio-economic							
Labour market	Retired	32,331	*56.35*	22,840	*51.78*	9491	*71.55*
	Employed or self-employed	15,726	*27.41*	14,348	*32.53*	1378	*10.39*
	Unemployed	1923	*3.35*	1621	*3.68*	302	*2.28*
	Sick or disabled	2091	*3.64*	1223	*2.77*	868	*6.54*
	Other	5301	*9.24*	4076	*9.24*	1225	*9.24*
Income quartile^a^	1st	14,499	*25.26*	10,437	*23.65*	4062	*30.61*
	2nd	14,689	*25.59*	10,736	*24.33*	3953	*29.79*
	3rd	14,680	*25.58*	11,557	*26.19*	3123	*23.54*
	4th	13,523	*23.56*	11,392	*25.82*	2131	*16.06*
Social participation							
Formal learning activities		6736	*11.74*	5879	*13.32*	857	*6.46*
Informal care provision		13,539	*27.76*	10,563	*27.58*	2976	*28.41*
Sport clubs		14,718	*25.65*	12,309	*27.90*	2409	*18.16*
Voluntary work		9132	*15.91*	7533	*17.07*	1599	*12.05*
Religious activity		7449	*12.98*	5705	*12.93*	1744	*13.14*

Own calculations based on SHARE data 2010–2011

Chi-square results are significant at the 0.05 level

^a^Adjusting for national differences in income distribution

### Variables

The dependent variable used in the analysis is participation in FLA, formulated by the question if an individual *has attended an educational or training course in the past* 12 *months*. The dependent variable is binary.

The measure of morbidity is based on a list of somatic diseases, which includes myocardial infarction; stroke or cerebrovascular disease; diabetes or high blood sugar; chronic pulmonary diseases, including pneumonia, emphysema and asthma; arthritis, including osteoarthritis and rheumatic disease; cancer or malignant neoplasm, including leukaemia and lymphoma (without minor skin cancers); gastric or duodenal ulcer; Parkinson’s disease; cataracts; and hip, femoral and other types of fracture. Two groups are defined: individuals reporting less than two diseases (‘no multimorbidity’ group) and individuals reporting two or more diseases (‘multimorbidity’ group). This definition of multimorbidity is often used as an indicator of poor health in studies of behavioural consequences of poor health (Van der Akker et al. 2001; Huisman et al. 2003; Marengoni et al. 2011).

The explanatory variables used in the analysis are the same for all groups of the sample: with and without multimorbidity and among those groups for working and not working. Those variables are:

- *Education* the level of education attained is the basic indicator of human capital used in the analysis. Such an approach is based on the concept of human capital measurement in some statistical studies (e.g. OECD—Hanson 2008). SHARE data include a variable for educational attainment corresponding to the ISCED-97 scale. For this analysis, it was transformed into a simple variable distinguishing three levels of education: primary, secondary and higher education.
- *Demographic factors* sex, age, marital status and household size. Based on the ordinal variable of birth year, three age groups were differentiated: 50–64, 65–74 and 75+. Households were categorised into three groups depending on size: single, two members and three or more members. Marital status was categorised into single, married, divorced and widowed.
- *Socio*-*economic status* was measured by level of income. Income quartiles were calculated separately for each country and aggregated for all analysed countries.
- *Functional limitations and mental health problems* were represented by three variables: ability to perform the selected activities for functional status assessment, mental health problems and the occurrence of dementia. Selected activities to assess functional status include walking 100 m; sitting for about 2 h; getting up from a chair after sitting for a long period, climbing several flights of stairs without resting; climbing one flight of stairs without resting; stooping, kneeling or crouching; reaching or extending arms about shoulder level; pulling or pushing large objects like a living room chair; lifting or carrying weights over 10 lb/5 kilos, like a heavy bag of groceries; picking up a small coin from the table. Mental health is assessed using the Euro-D scale where poor mental health is assessed in case of occurrence of more than three symptoms (Prince et al. 1999). Additionally, a dichotomous variable for reporting dementia is created.
- *Caregiving and social participation* was based on two original binary variables of providing care inside or outside the household. These two activities were combined into one variable of providing any type of care. Social participation was defined as a set of binary variables: volunteering, participating in clubs or sports, and religious activity.

### Assessment methods

The multivariate analysis was based on a country-pooled logistic regression model: (1) with the grouping variable of morbidity and (2) with the grouping variable of morbidity and labour market activity. Average marginal effects were calculated and compared between morbidity groups (Allison 1999). Estimations were made separately for the multimorbidity and no morbidity groups as well as for the working and non-working respondents. An estimate in one group was considered to be different (stronger or weaker) from that in another group if it was outside the confidence intervals (1.96*SE) of the other group. Regression models controlled for country effects.

## Results

### Descriptive data on formal learning activities

Comparing FLA at an older age among groups of individuals with and without multimorbidity, it is observed that in the healthier group learning activities were nearly twice as large as in the group of individuals with multimorbidity (13.42 vs. 6.46 %, Table 1). Descriptive analysis indicated that FLA in the total sample were less common with increasing age, among individuals with disability and dementia, individuals who were single or widowed and individuals with poor human capital (primary education). Participation in formal learning was especially high among people with a higher education and those still working and increased with income (Table 2). In case of females, married and coming from extended households, learning participation in the healthier group was more than twice as big as in the group with poor health. Only unemployed respondents were more active in learning courses in the group with poor health than in the group without multimorbidity.

**Table 2:** Participation in formal learning activities at an older age: total sample and by morbidity status (unweighted)

Variables	Categories	Total sample	Less than 2 morbidities		2 morbidities or more	
		%	%	*p* value*	%	*p* value*
1 ADL+		4.92	6.49	*0.000*	3.62	*0.000*
Poor mental health		9.73	11.95	*0.000*	5.40	*0.000*
Dementia		2.00	2.18	*0.000*	1.80	*0.000*
Gender	Male	10.31	11.52	*0.000*	6.01	*0.075*
	Female	12.83	14.75		6.78	
Demographics						
Age	50–64	17.40	18.30	*0.000*	12.05	*0.000*
	65–74	8.05	8.76		6.00	
	75+	3.03	3.53		2.29	
Marital status	Single	13.81	14.78	*0.000*	10.05	*0.000*
	Married	12.14	13.57		6.65	
	Divorced	16.96	18.86		10.84	
	Widowed	6.04	7.46		3.56	
Household size	Single	9.99	11.81	*0.000*	5.96	*0.023*
	2 persons	10.95	12.32		6.32	
	3 persons+	15.10	16.60		7.70	
Human capital						
Level of education	Primary	4.06	4.69	*0.000*	2.48	*0.000*
	Secondary	12.12	13.27		7.71	
	Higher	26.83	28.57		17.99	
Socio-economic						
Labour market	Retired	6.05	6.86	*0.000*	4.10	*0.000*
	Employed or self-employed	26.04	26.22		24.17	
	Unemployed	12.48	12.03		14.90	
	Sick or disabled	5.07	5.31		4.72	
	Other	6.38	7.09		4.00	
Income quartile^a^	1st	6.01	6.90	*0.000*	3.72	*0.000*
	2nd	8.87	10.10		5.54	
	3rd	12.40	13.85		7.01	
	4th	20.28	21.72		12.58	
Social participation						
Informal care provision		16.98	19.02	*0.000*	9.74	*0.000*
Sport, clubs		21.68	22.94	*0.000*	15.23	*0.000*
Voluntary work		25.13	25.99	*0.000*	21.08	*0.000*
Religious activity		15.95	17.62	*0.000*	10.49	*0.000*
Country						
Denmark		21.64	22.69	*0.000*	15.43	*0.000*
Switzerland		20.89	22.18		13.09	
Netherlands		19.15	20.41		10.32	
Sweden		19.07	20.12		13.69	
Belgium		18.79	21.18		11.01	
Austria		13.01	13.68		10.43	
Estonia		11.98	15.32		5.54	
Germany		11.54	13.29		5.14	
France		9.73	11.11		4.96	
Czech Republic		9.14	10.19		5.93	
Slovenia		7.59	7.90		6.26	
Portugal		6.86	7.70		4.44	
Spain		6.62	7.54		3.86	
Hungary		5.64	7.43		2.16	
Italy		2.22	2.56		1.02	
Poland		2.22	2.75		0.50	

Own calculations based on SHARE data 2010–2011

Chi-square results are significant at the 0.05 level

** *p*-values for Chi-square tests of association between the dependent variable (learning activity) and other characteristics between multimorbidity groups

^a^Adjusted for country differences in income distribution

### Examination of the role of predictors based on multivariate analysis

Multivariate analysis of the predictors of FLA by number of morbidities (Table 3) indicated that females were significantly more likely to be involved in FLA than men. Note, that this gender difference was smaller in the multimorbidity group. Additionally, increasing age was negatively associated with learning activity, although again in the case of multimorbidity, the relation was much weaker. Marital status and household composition were insignificant when it came to involvement in FLA.

**Table 3:** Comparison of predictors (AMEs, SE) of older peoples’ formal learning activities depending on morbidity

Variables	No multimorbidity (<2 diseases)	Multimorbidity (≥2 diseases)
	AME (SE)	AME (SE)
1 ADL+ (ref. no ADL)	−0.003 (0.005)	−0.001 (0.002)
Poor mental health (ref. good mental health)	0.000 (0.003)	0.000 (0.003)
Dementia (ref. no dementia)	−0.042 (0.023)	−0.017 (0.013)
Female (ref. male)	0.030*** (0.003)	0.010** (0.003)
Age (ref. 50–64)		
65–74	−0.009 (0.005)	0.006 (0.004)
75+	−0.034*** (0.007)	−0.014** (0.005)
Marital status (ref. single)		
Married	0.001 (0.007)	−0.017 (0.010)
Divorced	0.018 (0.010)	−0.004 (0.007)
Widowed	0.021 (0.012)	−0.010 (0.007)
Household size (ref. single)		
2 persons	−0.010 (0.008)	−0.001 (0.007)
3 persons+	−0.003 (0.007)	0.004 (0.007)
Education (ref. primary)		
Secondary	0.047*** (0.005)	0.012*** (0.005)
Higher	0.129*** (0.009)	0.043*** (0.009)
Labour market position (ref. retired)		
Employed or self-employed	0.083*** (0.006)	0.063*** (0.011)
Unemployed	0.048*** (0.013)	0.055** (0.019)
Sick or disabled	−0.016 (0.011)	0.001 (0.008)
Other	0.008 (0.009)	0.005 (0.008)
Income (ref. 1st quartile)		
2nd quartile	0.008 (0.006)	0.008 (0.006)
3rd quartile	0.017** (0.006)	0.006 (0.006)
4th quartile	0.031*** (0.006)	0.014* (0.007)
Informal care giving (ref. no caregiving)	0.022*** (0.004)	0.002 (0.004)
Sports, clubs (ref. no club activity)	0.035*** (0.004)	0.024*** (0.005)
Volunteering (ref. no volunteering)	0.054*** (0.005)	0.042*** (0.007)
Religious activity (ref. no religious act.)	0.029*** (0.006)	0.014** (0.006)

The model controls for country. Own calculations based on SHARE data

*AME* average marginal effects, *SE* standard error

* *p* < 0.05, ** *p* < 0.01, *** *p* < 0.001

The analysis showed the importance of educational level attained for undertaking learning courses at an older age. Higher educational attainment was significantly, positively related to engaging in learning and increased the probability of formal learning activity in both multimorbidity groups, but was much stronger in the healthy group. Being active in the labour market increased the probability of involvement in learning activities, with associations of similar strength irrespective of multimorbidity.

Higher incomes were another important factor increasing the chances of taking up learning activities. The effect of income was weaker in the multimorbidity group.

Engaging in other types of social activities, such as volunteering and religious activities, also increased the probability of being involved in formal learning. Informal care provision was significantly related to taking up learning courses, but only in the case of no multimorbidity. Each indicator of social participation showed a somewhat weaker association with learning activity in the multimorbidity group.

In summary, the predictors of learning activities between multimorbidity groups were similar. The strength of most predictors, however, is weaker in the multimorbidity group. As an exception, labour market activity is an equally strong predictor in both health groups.

The next step of the analysis was to compare predictors of participation in learning courses at an older age not only between multimorbidity groups, but also depending on labour market activity. Four groups were differentiated: labour market active without multimorbidity, labour market active with multimorbidity, labour market inactive without multimorbidity, and labour market active with multimorbidity. A comparison of the results between morbidity groups and across labour market activity indicated some important differences (Table 4).

**Table 4:** Comparison of predictors (AMEs, SE) of older peoples’ formal learning activities depending on morbidity and labour market activity

Variables	Labour market active: employed, self-employed, unemployed		Labour market inactive: retired, sick or disabled, other	
	No multimorbidity (<2 diseases)	Multimorbidity (≥2 diseases)	No multimorbidity (<2 diseases)	Multimorbidity (≥2 diseases)
1 ADL+ (ref. no ADL)	0.025 (0.014)	0.005 (0.019)	−0.006 (0.004)	−0.003 (0.002)
Poor mental health (ref. good mental health)	0.005 (0.008)	−0.002 (0.023)	−0.005 (0.003)	−0.004 (0.003)
Dementia (ref. no dementia)	−0.041 (0.116)	–	−0.054** (0.016)	−0.010 (0.011)
Female (ref. male)	0.067*** (0.007)	0.032 (0.023)	0.029*** (0.003)	0.008** (0.003)
Age (ref. 50–64)				
65–74	−0.045* (0.015)	−0.091** (0.029)	−0.053*** (0.003)	−0.024*** 0.003
75+	−.108* (0.037)	−0.029 (0.085)	−0.072*** (0.003)	−0.039*** (0.004)
Marital status (ref. single)				
Married	−0.028 (0.016)	0.009 (0.050)	−0.020** (0.007)	−0.012 (0.007)
Divorced	0.002 (0.018)	0.059 (0.065)	0.008 (0.008)	0.000 (0.007)
Widowed	−0.021 (0.024)	0.009 (0.075)	−0.005 (0.008)	−0.009 (0.006)
Household size (ref. single)				
2 persons	−0.021 (0.018)	−0.029 (0.048)	−0.015* (0.006)	−0.007 (0.006)
3 persons+	0.004 (0.019)	0.013 (0.050)	0.003 (0.007)	−0.000 (0.006)
Level of education (ref. primary)				
Secondary	0.139*** (0.012)	0.072* (0.032)	0.061*** (0.004)	0.023*** (0.004)
Higher	0.291*** (0.014)	0.242*** (0.043)	0.159*** (0.005)	0.075*** (0.010)
Income quartile (ref. 1st)				
2nd quartile	0.036* (0.016)	0.026 (0.041)	0.021*** (0.005)	0.013* (0.005)
3rd quartile	0.071*** (0.015)	0.028 (0.039)	0.038*** (0.005)	0.017** (0.006)
4th quartile	0.099*** (0.014)	0.068 (0.039)	0.070 (0.006)	0.034*** (0.008)
Informal care giving (ref. no cg)	0.060*** (0.008)	0.010 (0.026)	0.023*** (0.003)	0.003 (0.003)
Sports or clubs (ref. no clubs)	0.084*** (0.008)	0.083** (0.028)	0.043*** (0.004)	0.028*** (0.004)
Volunteering (ref. no vol.)	0.107*** (0.011)	0.192*** (0.035)	0.052*** (0.004)	0.046*** (0.006)
Religious activity (ref. no rel. act.)	0.046 (0.013)	0.038 (0.036)	0.023*** (0.004)	0.011* (0.004)

The model controls for country. In the case of the working population with multimorbidity, dementia is omitted due to collinearity. Own calculations based on SHARE data 2010–2011

*AME* average marginal effects, *SE* standard error

* *p* < 0.05, ** *p* < 0.01, *** *p* < 0.001

A negative effect of age on participation in formal learning was observed for both the labour market active and inactive, showing a decrease in the probability of taking up FLA, with a steeper gradient in the group without multimorbidity.

Marital status and household size now showed a significant association with formal learning activity in those who were inactive on the labour market. The coefficient was negative, indicating that single older people were more likely to participate in learning activities.

Among the factors increasing the likelihood of FLA within the older population were again human capital elements: previous educational attainment and its close correlate—income level. These relations were observed for both morbidity groups and despite labour market activity, with the effect of education being stronger for the labour market active.

Dementia was negatively associated with participation in learning courses among the labour market inactive. Other health variables were not significant in any group.

Social participation was again positively associated with participation in formal learning. The associations of leisure activity and volunteering with participation in learning were particularly strong for the labour market active group, while informal care provision and religious participation were insignificant. In the labour market inactive group, on the other hand, religious participation was significant in people with and without multimorbidity.

## Discussion

This study examined the probability of participation in FLA of older people in European countries, depending on health condition, as measured by the occurrence of multimorbidity, and distinguished by labour market activity. The predictors of learning activities between multimorbidity groups were similar. The strength of most predictors, however, was weaker in the multimorbidity group. In case of predictors of learning depending on health and labour market activity it was indicated that the negative gradient is more sound in the group of labour market inactive, who are typically older, and in poor health.

The learning activities of older people in the SHARE database are defined in a general sense by one item indicating participation in a learning course. The question of participation in learning activities does not include self-education and informal form of learning. Participation in formal activities performed outside the household, together with other participants is a typical social integration indicator important for the active and successful ageing concepts. However, as older people and people in poorer health often prefer more flexible and personalised, less formal activity (Withnall 2002; Holford et al. 2008), the use of this variable might be a limitation of the analysis. A study of informal learning would require richer data than obtained from the SHARE survey.

In this study, multimorbidity (having two or more diseases) was defined as a grouping variable to assess if it is a critical factor for learning activities. Our study did not take into account which diseases (or combination of diseases—Sinnige et al. 2013) would be critical for learning or the severity of the given diseases. However, the analyses control for functional limitation and mental disorders which may be considered as severity measures. We have examined whether older people despite suffering from multiple diseases, nevertheless have some potential for activity as SOC theory suggest (Baltes et al. 1999) and which resources are crucial for participation. Such an approach allows for a guarded interpretation of the results in relation to persons with multimorbidity.

Human capital measured by the level of education was found to be the strongest predictor of participation in learning courses in those without multimorbidity. Generally, positive relations between education and good health have been shown previously (e.g. Tuckett and Sargant 1999; Groot and van den Brink 2004; Mitchell 2007; Silles 2009). This study indicates that the relation is also significant for older people in the case of multimorbidity, albeit weaker. This might point to the idea that higher human capital is an elementary resource allowing an individual to overcome the negative effects of poor health and to stimulate social involvement. The relation between human capital and further learning is also stronger for the labour market active at an older age when compared to the inactive, which might be attributable to the fact that individuals who are still working are more involved in learning in the form of vocational courses and, for this group, morbidity level is of secondary importance.

The sample of older people included in the SHARE database has a wide age range, as it covers individuals aged 50 years or more. Our study confirms the previous findings that learning activity decreases with age (Heisel et al. 1981; Tuckett and Aldrige 2009; OECD 2013), but adds to these previous findings that the effect of age is weaker for individuals with multimorbidity. This suggests that a poor health condition might be of greater importance for learning potential than older age itself and might create additional barriers.

In contrast to the life-long learning of the labour market active, where men have been shown to be typically more involved in training than women (Chłoń-Domińczak and Lis 2013), older women in the labour market active group were more involved in learning than men. A comparison between morbidity groups shows that the higher involvement of females is visible also in the case of multimorbidity, although the effect is smaller for this group.

Although the literature provides evidence of other types of social involvement being obstacles to taking up learning activities due to opportunity costs (Withnall 2002), this study does not support these findings. Almost each type of social activity studied, volunteering, participating in sports and clubs, and religious activity, appeared to be positively associated with learning. In fact, some types of learning activities might be institutionally related to other types of social activity, such as club meetings or sport activities. The exception to the positive correlation of social activities and learning is provision of care, where multimorbidity might be an obstacle to both provision of care and learning. Additionally, religious participation proved not an important predictor of learning for the labour market active, but appeared important for the labour market inactive. Opportunity costs might play a role, as labour market active individuals might be less involved in the life of the religious community itself.

Presented analysis is based on cross-sectional data (SHARE wave 2010/2011). The defining feature of a cross-sectional study is that it allows to compare different population groups at a single point in time. The history of these groups that would allow for the analysis identification of developments or changes in their characteristics^1^ remains unknown. Although the SHARE survey has been conducted for almost a decade (2004–2013) and a part of the study is a panel data, it is still an insufficient period of observation for longitudinal, cohort analysis, especially as not all countries participated in all waves and questions on some areas of activity (including learning) have been reformulated. In the previous waves (2004, 2006) the question on participation in learning activities concerned a month, not 12 months preceding the survey (in 2010/2011).

## Conclusions

The FLA of adults are strongly determined by the level of education reached earlier in life. Also, life-long learning participation in courses among adults is higher for those active on the labour market. When moving into retirement FLA are still related to education level, however the effect of education decreases. At this stage of life, other factors predicting FLA emerge, including personal interest (leisure) and religious participation.

Promoting FLA for the older population is important for social integration and diseases prevention, but it should be kept in mind that the target population is characterised by multimorbidity, frailty, and often limitations in functional abilities. This population might not be healthier due to participation in learning courses, but its quality of life could be higher because of it and the ageing process could be more ‘successful’ or ‘happy’ (Smith 2005, 2013).

Another important policy-oriented conclusion concerns the typical social participation activities of older people, such as leisure activity and volunteering. Stimulating these activities might also bring positive effects to participation in formal learning.

Finally, it should be noted that participation in learning courses of older people in European countries depend not only on the individual factors analysed in the paper, but also on macroeconomic and institutional factors, including employment rate, welfare services, corporate responsibility for human capital investments via life-long learning during the cycle of a professional career, and local governments’ capabilities to integrate older people. The importance of such macro-factors may be high, especially for the population characterised by multimorbidity.

## Footnotes

1 For example American studies on baby boomers (born after the WW II) compared various cohorts of older generations what is possibly based on longitudinal data, bring a lot of information concerning changes in life style as well as health-related behaviours (Mitchel 2007; Martin et al 2009; The Gerontologist Special Issue 2012).
